# Influence of PWHT Parameters on the Mechanical Properties and Microstructural Behavior of Multi-Pass GTAW Joints of P92 Steel

**DOI:** 10.3390/ma15124045

**Published:** 2022-06-07

**Authors:** Sachin Sirohi, Amit Kumar, Shiva Soni, Gaurav Dak, Sanjeev Kumar, Aleksandra Świerczyńska, Grzegorz Rogalski, Dariusz Fydrych, Chandan Pandey

**Affiliations:** 1 Department of Mechanical Engineering, SRM Institute of Science and Technology, Delhi NCR Campus, Modinagar 201204, India; sachinsirohi2008@gmail.com (S.S.); sanjeevsrmmech@gmail.com (S.K.); 2 Department of Mechanical Engineering, Indian Institute of Technology Jodhpur, N.H. 62, Nagaur Road, Jodhpur 342037, India; kumar.175@iitj.ac.in (A.K.); kumar.143@iitj.ac.in (G.D.); 3 Department of Computer Science Engineering, SRM Institute of Science and Technology, Delhi NCR Campus, Modinagar 201204, India; shivasoni90@gmail.com; 4 Faculty of Mechanical Engineering and Ship Technology, Institute of Manufacturing and Materials Technology, Gdańsk University of Technology, Gabriela Narutowicza Street 11/12, 80-233 Gdańsk, Poland; aleksandra.swierczynska@pg.edu.pl (A.Ś.); grzegorz.rogalski@pg.edu.pl (G.R.)

**Keywords:** P92 steel, GTAW, heat treatment, impact toughness, hardness, tensile properties

## Abstract

The 9% Cr steels were developed for ultra-supercritical (USC) power plants to meet the requirements of high operating temperature and pressure. These steels are produced to operate at high temperatures where impact toughness is not a concern; however, it becomes important for the welded joints to have good impact toughness at room temperature for manufacturing. The present work investigates the effect of the post-weld heat treatment (PWHT) parameters, i.e., temperature and time, on the impact toughness of multi-pass gas tungsten arc welded (GTAW) joints of ferritic/martensitic grade P92 steel. The microstructural evolution in welded joints given varying post-weld temperatures and times was studied. The lath martensitic structure of the weld metal for the as-welded joints resulted in high hardness and low impact toughness. The weld fusion zone toughness was 12 J, which was lower than the minimum specified values of 41 J (ASME standards) and 47 J (EN ISO 3580:2017). The PWHT temperature and time were found to have a significant effect on the impact toughness of the weld metal. A drastic increase in the impact toughness of the weld metal was noticed, which was attributed to lath break-up, reduction in dislocation density and reduction in solid solution hardening. The maximum impact toughness of 124 J was measured for PWHT temperature and time of 760 °C and 120 min, respectively. The effect of PWHT parameters on tensile strength was also investigated, and test results showed that the joint was safe for USC boiler application as it failed from the region of the P92 base metal. The variation in microstructural evolution along the weldments resulted in hardness variation. PWHT led to homogeneity in microstructure and, ultimately, reduction in hardness value. According to the study, the optimum temperature and time for PWHT of a GTAW joint of P92 steel were found to be 760 °C and 120 min, respectively.

## 1. Introduction

To fulfill the growing demand for pollution-free electricity, ultra-supercritical (USC) power plants are in the development stage [1]. USC plants are designed to operate at an efficiency of approximately 45% with a working temperature of approximately 600–650 °C [2]. However, the major issue is producing a material that can operate at such high temperatures, as materials undergo considerable microstructural changes and degradation in mechanical properties, including oxidation, hydrogen-assisted cracking, embrittlement, and the evolution of inter-metallic phases [3,4,5,6,7]. To meet the demand for higher operating temperature and pressure in ultra-supercritical (USC) power plants, creep strength enhanced ferritic/martensitic (CSEF/M) steel having Cr, Mo and W as major alloying elements was developed [8,9,10]. In the CSEF/M steel family, P91, P911 and P92 steels were produced with a Cr content of approximately 9% and varying percentages of W, Mo, V and Nb [11]. P92 steel is an advanced member of the CSEF/M steel family and is produced to operate at a temperature of approximately 620 °C; beyond that, poor oxidation resistance limits its application [12]. P92 steel possesses a ferritic and martensitic microstructure that is derived from the normalizing and tempering process. The creep strength and high-temperature mechanical properties of P92 steel are derived from the martensitic microstructure and the controlled distribution of carbide [M(Cr, Fe, W, Mo)_23_C_6_, M(Cr, Mn, Fe)_7_C_3_, M(V, Nb)C] and nitride [M(V, Nb)N] precipitates [13,14]. The W and Mo contents also enhance the strength of P92 steel by imparting solid solution strengthening [15].

For power plant industries, the joining of P92 steel plates or pipes is mainly performed using fusion welding or high-energy beam welding processes [16,17,18]. Weldments of P92 steel offer poor mechanical properties at room temperature due to the formation of brittle fresh martensite in the weld fusion zone (WFZ). Other factors, including heterogeneous microstructure formation along weldments, the evolution of residual stresses along weldments, the formation of soft δ ferrite patches, the presence of diffusible hydrogen and the hardening of coarse-grained HAZ are also considered major causes of failure in P92 weldments [16,19,20,21,22,23,24,25]. Heterogeneity in the microstructure as a result of high heat input mainly leads to variation in mechanical properties, i.e., hardness and impact toughness. Failures of welded joints during service conditions are mainly reported in the soft region (poor hardness zone, i.e., inter-critical HAZ) and referred to as type IV failures [26,27]. The formation of brittle fresh martensite in the WFZ is mainly responsible for poor impact toughness [28]. Ferrite stabilizers such as Cr and W mainly promote the formation of soft ferrite patches in the P92 WFZ, which is also considered a major factor responsible for poor impact toughness [29,30]. For GTAW autogenous welded joints of P92 steel, the impact toughness of the WFZ was reported to be 3 ± 4 J, which was much lower than that of the P92 base metal (BM) or the recommended value of 47 J [31]. Saini et al. [32] also studied the role of filler composition on the impact toughness of shielded metal arc-welded joints of P91/P92 steel and observed poor values of 8 ± 2 J and 5 ± 3 J for P91 and P92 fillers, respectively. Arivazhagan et al. [33] studied the effect of varying Nb and V content on the impact toughness of P91 weldments and reported an adverse effect of V and Nb content on impact toughness. Sam et al. [29] studied various empirical relations to estimate the delta ferrite content in reduced activation ferritic martensitic (RAFM) steel weldments and proposed the Newhouse formula to evaluate the accurate value. The soft ferrite zone of hardness 190–210 HV also imparts heterogeneity to the weld microstructure, as the surrounding matrix of the delta ferrite is martensitic with hardness 400–500 HV in the as-welded condition [29,34]. The retained delta ferrite in WFZ also results in poor creep strength of the P92 welded joint [35]. The effect of varying diffusible hydrogen content on the mechanical behavior of P91 welded joints was also studied, revealing a negative impact of diffusible hydrogen content on mechanical properties [36,37]. The high heat input also resulted in variation in hardness along the weldments. The peak hardness was mainly measured in the WFZ and CGHAZ regions, due to the formation of fresh martensite, and was in the range of 400–500 HV [18,38]. However, the ICHAZ region showed poor hardness of 200–225 HV [18,38]. The great difference in hardness value between CGHAZ and ICHAZ also resulted in poor performance of the welded joint. Residual stress evolved in P92 weldments mainly due to the high heat input or the transformation of FCC austenite into BCT martensite during the cooling cycle of the welding process [39,40]. The residual stress present in the WFZ mainly combined with the crack-susceptible martensitic microstructure or the diffusible hydrogen present in the WFZ, leading to cracking in the weldments [41,42].

Hence, to meet the essential service condition, proper control of the amount of δ ferrite in the WFZ, a lower magnitude of residual stress, a lower amount of diffusible hydrogen in the WFZ, and uniformity in microstructure and mechanical properties along the weldments are all required. The composition of the WFZ can be altered to control delta ferrite formation. The weight percentages of W, Mo, V and Nb in filler metal can be optimized to minimize the delta ferrite content in the WFZ. The cooling rate also governs delta ferrite formation, and processes such as activated TIG (A-TIG) or autogenous TIG, having a higher cooling rate, promote the formation of delta ferrite in the WFZ. Sometimes, the adjacent HAZ, i.e., the CGHAZ, also shows the formation of delta ferrite patches due to the higher cooling rate [43]. The other factors, such as residual stress and heterogeneity in microstructure, can be minimized by using proper post-weld heat treatment (PWHT). The proper PWHT also improves the mechanical properties of the weldments [32]. Chalk [35] performed compositional modeling for a P92 welded joint and observed the effects of the individual elements on lower critical temperature. Initially, Ni and Mn weight percentage was considered for evaluating the A_1_ temperature and finalizing the PWHT temperature. Subsequently, Santella modified this procedure [44] for both P91 and P92 steel. Based on the composition modeling analysis performed by Chalk [35], the A_1_ temperature for any welding consumable is calculated by using the following Equation (1):

A_1_ = 809.7 − 99.5C − 196.4N − 67.4Mn − 78.8Ni − 9.85Co − 47.2Cu + 20B+10.9Si + + 35.8P + 95.6S + 3.7Cr + 19.7Mo + 31.4Nb + 53.8V + 10.6W(1)

The PWHT temperature was recommended to be between the A_1_ and A_c1_ temperatures of P92 steel (797 °C) [35]. In other published work, PWHT temperatures between 700–780 °C and times between 1–3 h were reported, depending on the chemical composition of the filler metal and the welding process. 

Saini et al. [38] studied the impact of PWHT on SMAW joint P92 steel. PWHT was performed at 760 °C for 2 h. Acceptable levels for the mechanical properties were obtained only after PWHT. The impact toughness of WFZ measured higher than the minimum recommended value of 47 J. PWHT at 760 °C for 2 h also helped to minimize heterogeneity in the microstructure and mechanical properties along the weldments. Pandey et al. [45] also reported PWHT at 760 °C for 2 h for an autogenous TIG joint of P92 steel to achieve an acceptable level of impact toughness and tensile strength. Seo et al. [46] studied the role of varying PWHT on the homogenization of the microstructure and mechanical properties in P92 HAZ for dissimilar weldments of IN740H/P92 steel, and recommended an optimum PWHT temperature of approximately 760 °C. To obtain a homogenized microstructure in P92 HAZ, Fei et al. [45] optimized the PWHT temperature for dissimilar A-TIG weldments of P92/austenitic steel. PWHT was carried out in a temperature range from 550 to 760 °C, and 760 °C was considered the most appropriate PWHT temperature. Wu et al. [47] also studied the effect of PWHT at 760 °C for 2 h on the mechanical behavior of P92 steel welded joints. PWHT was reported to have a drastic effect on microstructural evolution, which resulted in considerable improvement in the mechanical properties of the welded joint. The effect of PWHT time on the mechanical properties of P92 weldments was also investigated [48]. For autogenous joints, to obtain proper tempering of the martensite, PWHT time was recommended to be more than 2 h, while for multi-pass TIG joints, PWHT time was recommended to be approximately 2 hrs. However, PWHT was observed to have a minute effect on the morphology of the soft delta ferrite patches, and these remained in the microstructures of the WFZ and CGHAZ [49]. To eliminate soft ferrite in the WFZ, a normalizing-based tempering was recommended in a few recently published works [30,49]. A positive effect of PWHT on residual stress and creep strength of the welded joint was also reported [20,27,50,51,52].

From several studies reviewed, it was found that various researchers had studied the microstructural and mechanical behavior of similar and dissimilar P92 weldments. In most cases, welding was performed using the SMAW process and PWHT was carried out at 760 °C. Thus, a systematic study is required to identify the role of PWHT parameters in the microstructural and mechanical properties of multi-pass GTAW joints of P92 steel. This research work investigates the effect of varying PWHT parameters on mechanical, microstructural, and residual stress properties of GTAW joints of P92 steel. 

## 2. Materials and Methods

**Figure 1:**
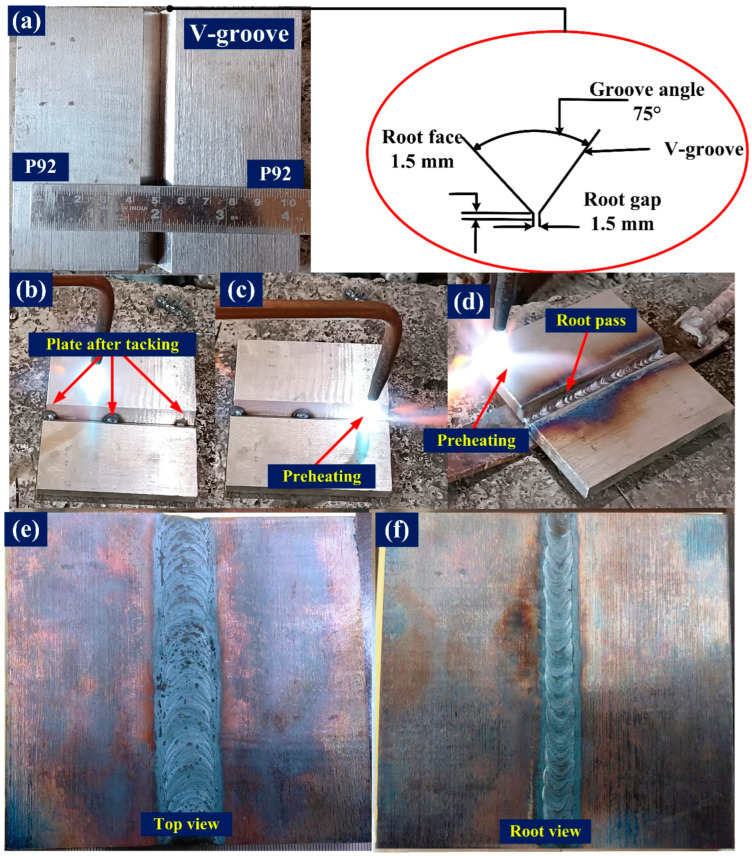
(**a**) Grooved plate and detail of groove geometry, (**b**) plate after tacking, (**c**) preheating, (**d**) root pass, (**e**) top view of plate, (**f**) root view of the plate.

P92 steel pipes with an outer diameter of 60 mm and a thickness of 11 mm were received in normalized and tempered conditions. Each pipe was cut into two halves and forged. Each forged P92 steel plate was normalized at 1040 °C for 10 min and tempered at 760 °C for 120 min to stabilize the microstructure and impart the essential mechanical properties. A plate of dimensions 150 mm × 65 mm × 10 mm was machined from each normalized and tempered (N&T) steel plate. The composition of the plate was analyzed using an optical emission spectrometer and is described in Table 1. A conventional V-groove geometry was machined at the edge of the plate, with a groove angle of 75° and root height of 1.5 mm (Figure 1a). Before welding, plates were tacked as shown in Figure 1b. Each plate was cleaned using acetone and preheated at a temperature of 280 °C before welding (Figure 1c). Multi-pass V groove butt joints were produced using a gas tungsten arc welding (GTAW) machine (Make: Lincoln (Dearborn, MI, USA), Model: Precision TIG 225), employing 2.4 mm diameter BOHLER P92-IG (P92) filler and a thoriated tungsten electrode (EW-Th-2) with diameter of 3.2 mm. Pure argon (purity: 99.995%) was used as shielding gas. Welding was performed in the flat position. The composition of the filler metal is depicted in Table 1. Welding was performed in four passes, including the backing and capping pass. The welding process parameters and heat inputs for the root pass, filling pass, capping pass, and backing pass are listed in Table 2. The heat input (per unit length) depends on the welding input parameters and can be expressed using Equation (2) [53],

(2) $$H=\frac{\eta \times V\times I}{S}$$

where *H* is heat input per unit length (J/mm), *I* is welding current (A), *V* is arc voltage (V), *S* is travel speed (mm/s) and *η* is arc efficiency (60% [54]).

The plate after the root pass is depicted in Figure 1d. The top and root views of the welded plate are shown in Figure 1e,f. After each pass, the weld area was cleaned using a wire brush. The inter-pass temperature was maintained at 280–300 °C. Commercially pure argon (99.9%) was used to shield the arc column and weld area and was supplied at a constant flow rate of 15 L/min. After completion of the welding, post-weld heat treatment (PWHT) was carried out for varying temperatures and times as shown in Table 3. Both sides of the joints were visually inspected, during which no welding imperfections were found.

**Figure 2:**
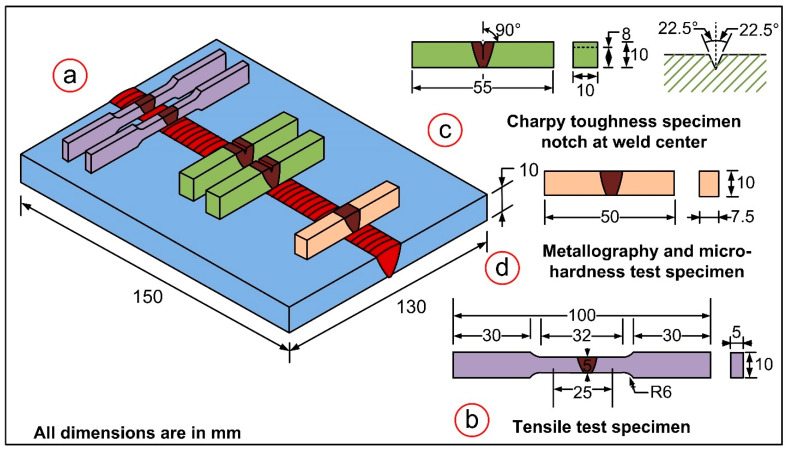
Schematic showing the (**a**) welded plate with specimen positions, (**b**) standard tensile specimen, (**c**) impact toughness specimen, (**d**) specimen machined for metallography and hardness testing.

Microhardness testing, room temperature tensile testing, and impact toughness testing were carried out to investigate the mechanical properties of the as-welded (AW) and PWHT joints. Each mechanical testing specimen was extracted from the welded plate as shown in the schematic (Figure 2a). The tensile test was performed on a standard specimen (Figure 2b) with a constant extension speed of 1 mm/min using a Vertical Tensile Testing Machine (Instron 5980 of 100 kN capacity, Instron, MA, USA). The fractured specimen was investigated using a scanning electron microscope. The room temperature impact toughness test was performed on a standard impact specimen (Figure 2c) of dimensions 55 mm × 10 mm × 10 mm, with a central V-notch in weld metal with a depth of 2 mm. The test was conducted on a Charpy Impact Tester (FIT-400-ASTM-D) according to the ASTM E23 standard [55]. The notch was machined in a direction parallel to the welding direction to ensure that the crack propagation path could pass through the capping, center and backing pass zones of the weld metal. For hardness measurement and microstructural characterization, a sample of dimensions 50 mm × 10 mm × 7.5 mm was machined from the welded plate (Figure 2d). Hardness distribution plots were taken along the transverse direction of the weldments using a Vickers Microhardness Tester (Mitutoyo, Autovick HM-200). The indentation was taken at a load of 1000× *g* for a dwell time of 10 s. The microstructure was studied at different weldment regions, including the weld metal, the heat-affected zone (HAZ) and the interface. Field Emission Scanning Electron Microscopy and an optical microscope (Leica, DMC4500, Leica Microsystems GmbH, Wetzlar, Germany) were used to reveal weldment microstructure. X-ray diffraction analysis of the P92 base metal was performed to detect the phases. The raw data obtained from XRD analysis were analyzed using X’pert High Score software.

## 3. Results and Discussion

### 3.1. As-Received Materials

**Figure 3:**
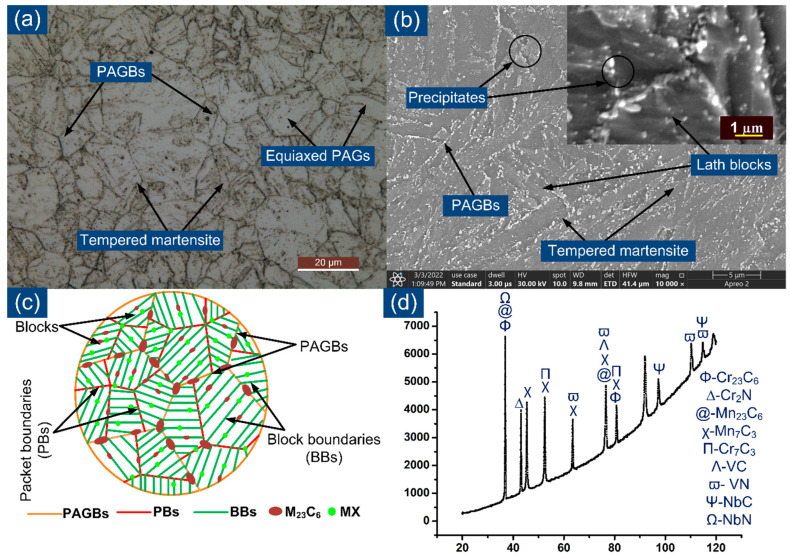
(**a**) Optical image showing tempered martensitic microstructure and boundaries, (**b**) SEM image showing lath blocks, boundaries and precipitate decoration along boundaries and inside the matrix, (**c**) schematic showing precipitate decoration along boundaries and blocks, and (**d**) XRD profile showing the phases present in P92 steel.

The micrograph of the P92 steel exhibited a tempered martensitic microstructure, as depicted in Figure 3a. Figure 3a shows equiaxed prior austenite grains (PAGs) with an average size of 12 ± 5 µm, lath blocks and boundaries. The SEM image, captured at a magnification of 10,000×, reveals the lath blocks, boundaries (block boundaries (BBs), packet boundaries (PBs) and prior austenite grain boundaries (PAGBs)) and decoration of the precipitates (Figure 3b). The precipitates located along the PBs and PAGBs are coarse in size and were confirmed to be Fe-, Mn- and Cr-rich M_23_C_6_ precipitates [56]. The size of the coarse precipitates was in the range of 100–200 nm. Other fine precipitates, with sizes lower than 50 nm, were also observed within the lath blocks and confirmed to be Nb- and V-rich carbonitrides (VC, VN, NbC and NbN) [56]. The evolution of the microstructure in N&T P92 steel is also represented by a schematic image, given in Figure 3c. The other precipitates, except M_23_C_6_ and MX, were M_7_C_3_ and Cr_2_N as confirmed via X-ray diffraction (XRD) analysis (Figure 3d). Hurtado-Noreña et al. [57] also reported the presence of M_23_C_6_, M_3_C and MX precipitates in 9% Cr steels. The tensile strength and yield strength of P92 after N&T treatment were 758 ± 6 MPa and 520 ± 8 MPa, respectively. 

### 3.2. Weldment Characterization

**Figure 4:**
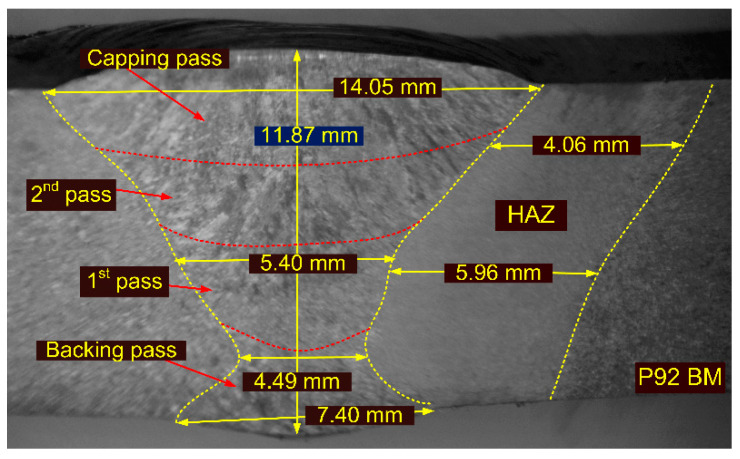
Macrograph of the welded joint.

The welding was completed in four passes and each pass is marked in the macrograph of the welded joint shown in Figure 4. The weld widths at the capping and backing passes were 14.05 mm and 7.40 mm, respectively. The widths of the HAZ corresponding to the capping pass and filling pass (second pass) were 4.06 mm and 5.96 mm, respectively. 

**Figure 5:**
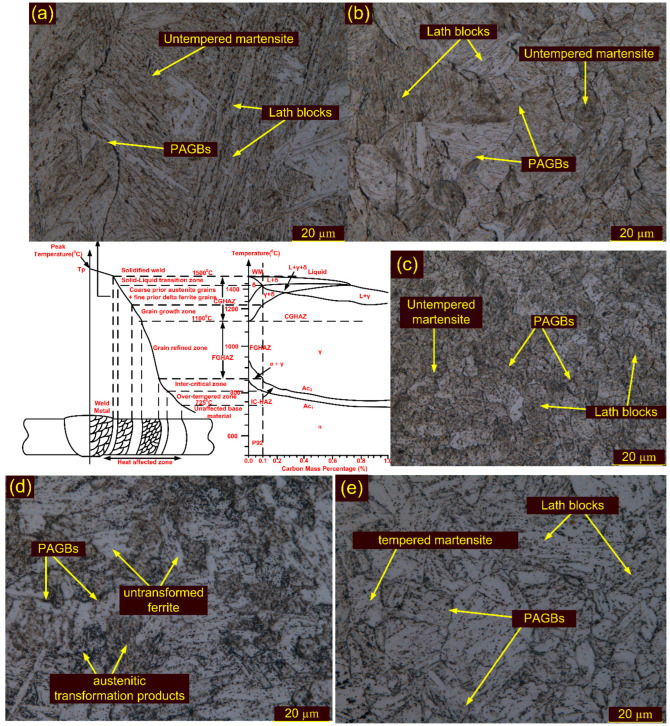
Fe–C diagram showing the formation of various zones in P92 weldments corresponding to different peak temperatures, and optical images of weldments: (**a**) WFZ, (**b**) CGHAZ, (**c**) FGHAZ, (**d**) ICHAZ, (**e**) SCHAZ.

The effect of the welding thermal cycle on microstructural evolution along weldments is depicted in a systematic Fe–Fe_3_C phase diagram (Figure 5). Figure 5 also presents the peak temperature experienced by each sub-zone and the corresponding optical micrographs. The microstructural evolution strongly influences the variation in mechanical properties along the sub-zones during the welding thermal cycle [58]. Zhang et al. [50] had reported the lower critical temperature (A_c1_) and upper critical temperature (A_c3_) for P92 steel to be 835–845 °C and 920–930 °C, respectively. The peak temperature experienced by the region varied as it moved away from the fusion boundary toward the BM. The weldment could be divided into five sub-zones on the basis of A_c1_, A_c3_ and peak temperature (Figure 5). The sub-zones will be referred to as the weld fusion zone (WFZ), coarse-grained HAZ (CGHAZ), fine-grained HAZ (FGHAZ), inter-critical HAZ (ICHAZ) and sub-critical HAZ (SCHAZ) [59].

**Figure 6:**
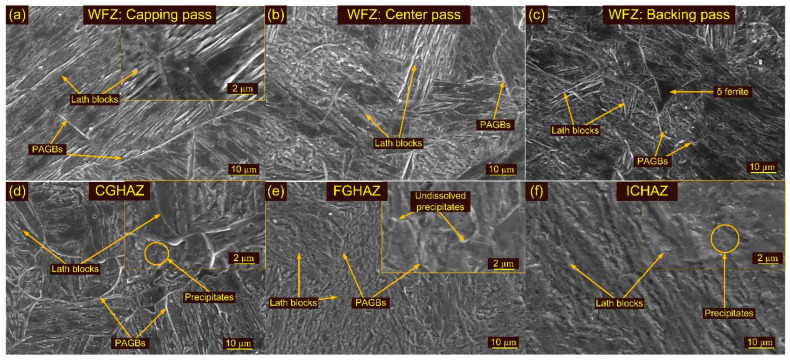
SEM images along weldments: WFZ corresponding to (**a**) capping pass, (**b**) center pass, (**c**) backing pass; (**d**) CGHAZ, (**e**) FGHAZ, (**f**) ICHAZ.

The micrograph of the WFZ consists of a typical columnar lath martensitic microstructure. The lath blocks and packet boundaries are seen very clearly in Figure 5a. The SEM image corresponding to the WFZ shows the complete dissolution of the precipitates, as the temperature was much higher than the melting temperature. The lath blocks of different orientations within the lath packets are marked in Figure 6a–c. The WFZ of P92 steel is more prone to δ ferrite formation because of the presence of Cr, W and Mo [60]. Based on the chemical composition of the WFZ (Table 1), various empirical relationships, i.e., the ferrite factor (*K_ff_*), have been suggested for evaluating δ ferrite formation. Schneider’s formula is the method most commonly used to predict the formation of δ ferrite [61]. The *K_ff_* and *Cr_eq_* are used to estimate ferrite formation and are listed in Equations (3) and (4):

(3) $$Cr_{eq}=\%Cr+6\%Si+4\%Mo+1.5\%W+11\%V+5\%Nb+12\%Al+8\%Ti-40\%C-2\%Mn-4\%Ni-2\%Co\;-30\%N-\%Cu$$

(4) $$K_{ff}=\%Cr+6\%Si+4\%Mo+5\%Nb+2\%Al+8\%Ti-40\%C-2\%Mn-4\%Ni-40\%N$$

**Figure 7:**
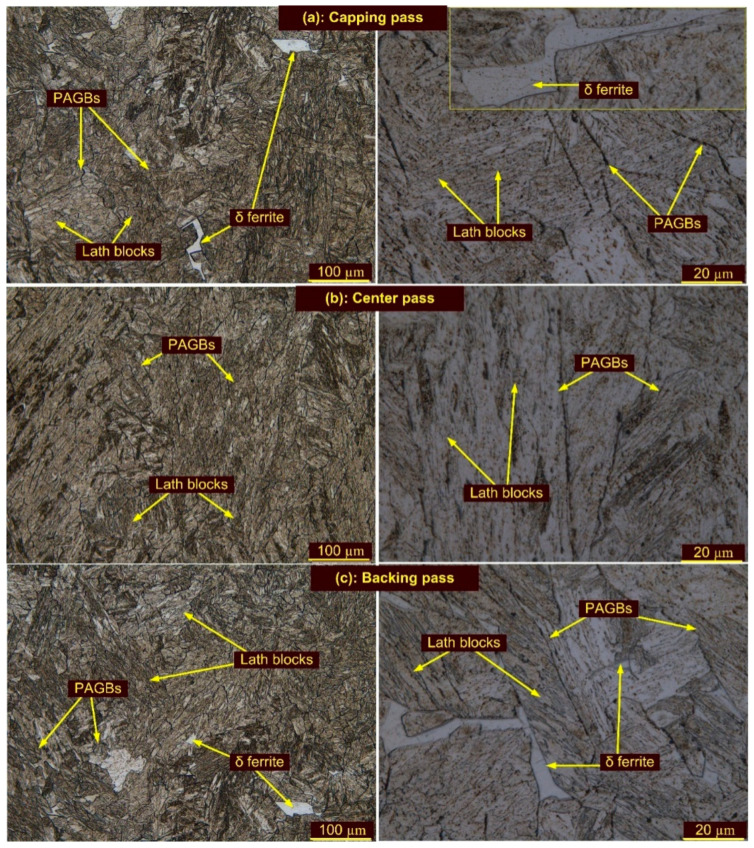
Optical image of WFZ corresponding to passes: (**a**) capping pass, (**b**) center pass, (**c**) backing pass.

In order to obtain a δ ferrite-free WFZ, *Cr_eq_* < 10 and *K_ff_* < 8. Based on the above equations and using Table 1, the values of *Cr_eq_* and *K_ff_* are 9.42 and 4.64, respectively. Both values were measured to be within limits, so no δ ferrite patches were expected to be present in the WFZ. However, δ ferrite patches were seen in the WFZ (Figure 7a–c) corresponding to the capping and backing passes, which might be due to variation in cooling rate, as in both passes the deposited metal was directly in contact with air. At higher magnification, the WFZ showed a few needle-shaped precipitates with lengths of 200–250 nm and spherical shape precipitates with sizes of 40–50 nm within the lath blocks (Figure 6a). The needle-shaped precipitates might be Fe-rich M_3_C, while spherical precipitates might be Nb-rich MX, as both precipitates showed higher thermal stability [57,62,63].

The region adjacent to the fusion boundary, i.e., CGHAZ, had a temperature much higher than A_c3_, which led to the dissolution of transgranular and inter-M_23_C_6_ precipitates that inhibit the movement of PAGBs. The dissolution of M_23_C_6_ precipitates made the boundaries free to grow and resulted in the formation of coarse PAGs, as depicted in Figure 5b. The CGHAZ exhibited the typical untempered lath microstructure, having coarse PAGs, lath blocks and boundaries (Figure 5). The PAGs’ size in the CGHAZ was 23 ± 4 µm. The SEM image of CGHAZ is depicted in Figure 6d and is characterized by lath blocks and boundaries. The magnified view of the CGHAZ shows the needle-shaped and spherical precipitates, which might be undissolved Fe-rich M_3_C and V and Nb-rich MX [47]. The FGHAZ adjacent to the CGHAZ was subjected to peak temperature just near or exceeding A_c3_ consisting of fine PAGs and an untempered martensitic microstructure (Figure 5c). Since the peak temperature in the FGHAZ region was lower than the recrystallization temperature, there was an absence of rapid grain growth. The temperature in the region was also not sufficient to dissolve the M_23_C_6_ precipitates, and these undissolved precipitates (Figure 6e) also inhibited the movement of grain growth. Hence, the grain growth in the FGHAZ was limited as compared to the CGHAZ, although a high peak temperature was experienced. The PAGs’ size was measured to be 12 ± 2 µm. The SEM image shows the presence of undissolved precipitates along the boundaries and blocks (Figure 6e). The next sub-zone adjacent to the FGHAZ is the ICHAZ, which experienced temperatures between A_c3_ and A_c1_, resulting in partial austenite transformation and negligible dissolution of the precipitates. The partial transformation of austenite resulted in a bimodal microstructure with both coarse and fine-grained regions along with untransformed ferrite (UF), i.e., tempered martensite, and austenite transformed product (ATP), i.e., fresh martensite (Figure 5d). The region subjected to the transformation of austenite behaved similarly to P92 BM in a normalized state. The ATP and UF in the ICHAZ are marked in the SEM image (Figure 6f). The decoration of the precipitates along the blocks and boundaries is depicted in a higher magnification SEM image (Figure 6f). The PAGs’ size was measured to be 10 ± 2 µm. SEM images of the FGHAZ (Figure 6e) and ICHAZ (Figure 6f) confirm that the distribution of the precipitates in the FGHAZ/ICHAZ was completely different from that in the P92 BM (Figure 3b) and the CGHAZ (Figure 6d). The SGHAZ region, between the unaffected BM and the ICHAZ, experienced a temperature lower than A_c1_ and exhibited an over-tempered martensitic microstructure (Figure 5e). In the SGHAZ, tempering of the martensite occurred; however, there was no phase transformation in this region [64]. Temperatures lower than A_c1_ sometimes lead to the coarsening of M_23_C_6_ precipitates. The size of PAGs was 15 ± 3 µm in the SGHAZ, which was similar to the P92 BM.

A micrograph showing the effect of the welding passes on the WFZ is given in Figure 7. The WFZ corresponding to the capping and backing passes showed a microstructure type with elongated grains and typical lath blocks (Figure 7a–c). For both passes, WFZ showed the presence of δ ferrite patches of different shapes and morphology. The WFZ corresponding to the backing and capping passes was directly exposed to the surroundings and achieved a higher cooling rate. Ferrite stabilizers such as W and Cr, present in the filler metal, also accelerated the formation of the ferrite patches in the WFZ. Previous studies also reported the role of ferrite stabilizer content on the formation of ferrite patches [62].

During the solidification of the weld metal, the δ ferrite (BCC) nucleates from the liquid at a temperature of approximately 1500 °C and the transformation is completed at approximately 1426 °C [30]. The transformation from δ ferrite to austenite (FCC) starts at a temperature of approximately 1426 °C and is completed at approximately 1200 °C. However, 100% transformation of the δ ferrite into austenite depends upon the cooling rate and ferrite content present in the weld metal. In the capping and backing passes, a fast cooling rate resulted in an incomplete transformation of the δ ferrite to austenite. That resulted in δ ferrite retained in the WFZ, corresponding to the backing and capping passes. The WFZ corresponding to the center pass (Figure 7b) showed a negligible amount of δ ferrite patches. The SEM image corresponding to the center pass also showed the absence of δ ferrite (Figure 6b). During the capping and backing passes, the WFZ corresponding to the center pass experienced temperatures above A_c1_ or below the melting temperature of BM, which resulted in δ ferrite dissolution and transformation into austenite, which transformed into martensite on further cooling. As per the report of Saini et al. [30], δ ferrite shows poor stability below 1250 °C. The WFZ region with δ ferrite patches showed the dissolution of δ ferrite via diffusion of the Cr and W when subjected to a temperature above the A_c3_ of P92 BM (887 °C) and below 1250 °C. A similar concept is applicable for the WFZ corresponding to the center pass. A higher-magnification micrograph of the WFZ corresponding to each pass shows the typical lath blocks and elongated PAGs. The δ ferrite patches are seen mainly at the PAGBs, due to increased free energy at the PAGBs than in the grain interior (Figure 7a–c). Due to the variation in free energy between the PAGBs and the grain interior, δ ferrite patches of various amounts and morphologies were formed in the martensitic matrix. The SEM image corresponding to the backing pass also shows the availability of δ ferrite patches (Figure 6c). The PAGs’ size in the capping, center and backing passes were 24 ± 5 µm, 36 ± 5 µm, and 28 ± 4 µm, respectively. The presence of coarse PAGs in the center pass might be due to the backing and capping passes’ reheating effect. 

**Figure 8:**
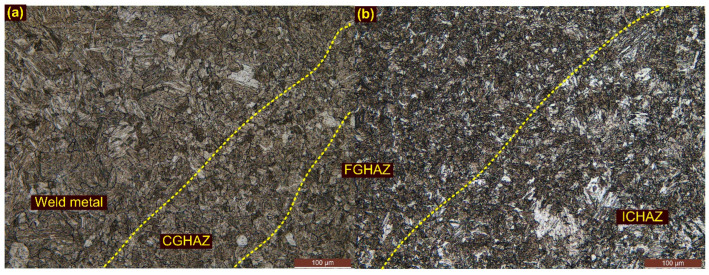
(**a**) Interface of the WFZ and CGHAZ; (**b**) interface of the FGHAZ and ICHAZ.

Figure 8 shows the boundary between the WFZ and the HAZs. Figure 8a represents the interface of the WFZ and the CGHAZ and ensures the proper mixing of the BM and the filler metal. Figure 8b shows the interface of the FGHAZ and the ICHAZ. A microstructural variation across the interface is very clearly observed.

**Figure 9:**
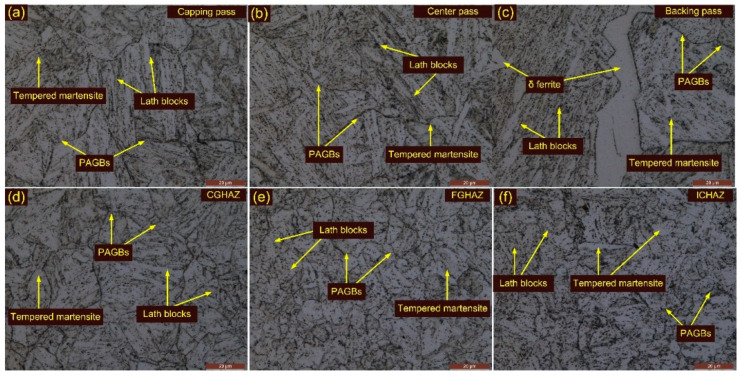
Weldments after T1 treatment: WFZ (**a**) capping pass, (**b**) center pass, (**c**) backing pass; (**d**) CGHAZ, (**e**) FGHAZ, (**f**) ICHAZ.

**Figure 10:**
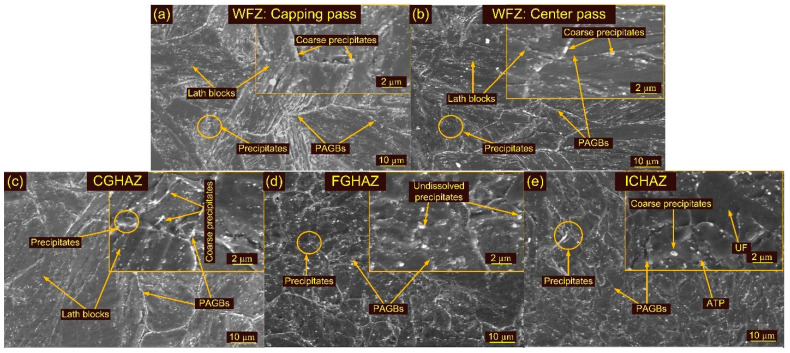
SEM images of weldments after T1 treatment: WFZ (**a**) capping pass, (**b**) center pass; (**c**) CGHAZ, (**d**) FGHAZ, (**e**) ICHAZ.

**Figure 11:**
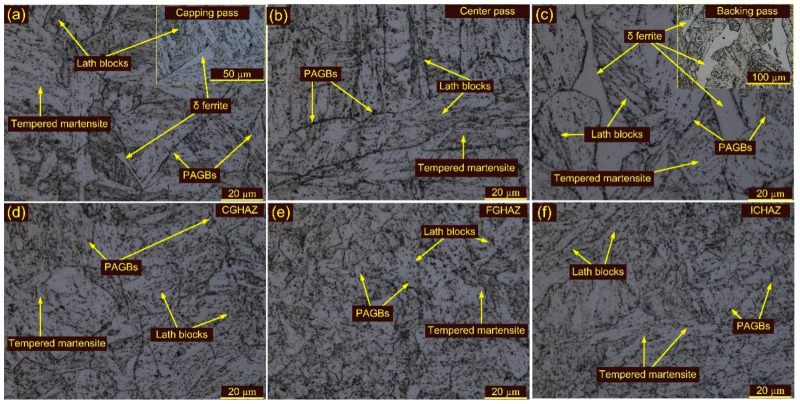
Weldments after T2 treatment: WFZ (**a**) capping pass, (**b**) center pass, (**c**) backing pass; (**d**) CGHAZ, (**e**) FGHAZ, (**f**) ICHAZ.

**Figure 12:**
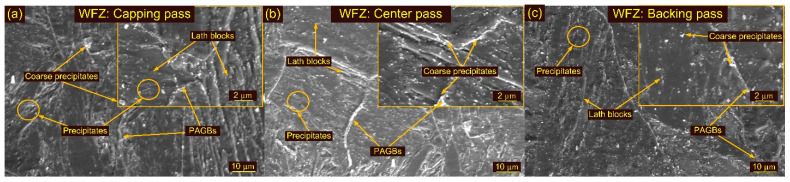
SEM images of weldments after T2 treatment: WFZ (**a**) capping pass, (**b**) center pass, (**c**) backing pass.

Optical images of the weldments after T1 are depicted in Figure 9a–f and their corresponding SEM images in Figure 10a–e. Optical images of the weldments after T2 are depicted in Figure 11a–f and their corresponding SEM images in Figure 12a–c. The T1 and T2 treatments resulted in a tempering of the martensite and the evolution of the precipitates in the WFZ. A tempered martensitic microstructure was seen in the WFZ for T1 (Figure 9a–c) and T2 (Figure 11a–c). Micrographs of the WFZ were captured in the capping, center and backing passes to observe the combined effect of PWHT and the welding passes. The δ ferrite patches observed in the WFZ for the AW joint also remained after PWHT. The WFZ corresponding to the backing pass (Figure 9c) and capping pass (Figure 11a,c) show the presence of δ ferrite patches. As mentioned by Saini et al. [30], dissolution of the δ ferrite mainly starts after heating to a temperature greater than the A_c3_, while in the present work, PWHT was carried out at 760–780 °C, which was lower than the A_c3_.

The PAGs’ sizes in the WFZ corresponding to the capping, center and backing passes were 25 ± 4 µm, 35 ± 5 µm, and 27 ± 3 µm, respectively, for T1 treatment, and 28 ± 3 µm, 38 ± 2 µm, and 30 ± 2 µm, respectively, for T2 treatment. From these results, it is clear that PWHT time (T1 and T2) had a minute or negligible effect on grain size.

The major phase that evolved in P92 steel weldments during the PWHT was M_23_C_6_, V(N,C) and Nb(C,N). The precipitates are seen clearly in the WFZ for both treatments (T1 and T2). The evolution of the precipitates mainly occurred along the BBs, PBs and PAGBs as marked in the SEM images (Figure 10a,b and Figure 12a–c). Higher magnification SEM images corresponding to the welding passes show the presence of both coarse and fine precipitates (Figure 10a,b and Figure 12a,c). The precipitates along the blocks and boundaries were coarse in nature and their size was measured in the range of 75–250 nm. Within the laths, fine precipitates with sizes of less than 50 nm were seen and confirmed as V- and Nb-rich carbides and nitrides [63].

The CGHAZ after T1 and T2 showed a complete change in microstructure due to the evolution of the precipitates and exhibits the tempered martensitic microstructure Figure 9d and Figure 11d. The PAGs’ size remained unaffected after T1, compared to the CGHAZ in the AW joint, and was 24 ± 6 µm. However, a noticeable increase in PAGs’ size was measured after T2 (32 ± 6 µm). Figure 10c shows the SEM image of the CGAHZ after T1 and confirms the evolution of the precipitates along boundaries, blocks and the matrix. This resulted in a uniform distribution of the precipitates along boundaries and the matrix. The coarse M_23_C_6_ precipitates evolved at the boundaries while fine MX evolved within the matrix during T1 and T2 treatments. The FGHAZ also showed a tempered martensitic microstructure; however, PAGs’ size was measured to be lower than in the CGHAZ. For T1 and T2, PAGs’ size in the FGHAZ was 13 ± 3 µm and 14 ± 1 µm, respectively. PAGs’ size after T1 and T2 remained constant, as compared to the FGHAZ of the AW joint. Figure 9e and Figure 11e show optical images of the FGHAZ corresponding to T1 and T2 welded joints. The SEM image after T1 (Figure 10d) shows nonuniformity in the size and distribution of the precipitates. This might be due to the simultaneous evolution of the new MX and M_23_C_6_ precipitates and coarsening of the existing undissolved M_23_C_6_ and MX precipitates. During welding, the MX precipitates remained undissolved in the FGHAZ and did not show any growth during T1. Hence, after T1, the major coarsening was seen for M_23_C_6,_ which ultimately contributed to the heterogeneity in the size and distribution of the precipitates, while Nb(C, N) and V(C, N) remained unaffected [46]. The heterogeneous microstructure of the FGHAZ made it susceptible to Type IV cracking. Figure 9f and Figure 11f show optical micrographs of the ICHAZ for T1 and T2, respectively. The ICHAZ did not show any major change after PWHT; however, there might be coarsening of the carbide precipitates. It shows a typical tempered martensitic microstructure with fine PAGs and randomly distributed precipitates. However, heterogeneity in the size of the precipitates might occur because of the coarsening of the carbide precipitates. A typical SEM image of the ICHAZ for T1 is shown in Figure 10e. It shows two different regions, i.e., UF and ATP, and the density of the precipitates along the boundaries of UF and ATP was different (Figure 10e). The precipitates that coarsened after treatment are also marked in Figure 10e. The PAGs’ size in the ICHAZ was 10 ± 2 µm and 12 ± 3 µm in UF and ATP, respectively.

**Figure 13:**
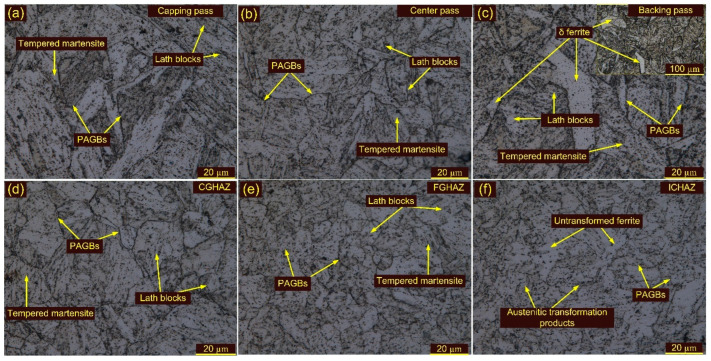
Weldments after HT1 treatment: WFZ (**a**) capping pass, (**b**) center pass, (**c**) backing pass; (**d**) CGHAZ, (**e**) FGHAZ, (**f**) ICHAZ.

**Figure 14:**
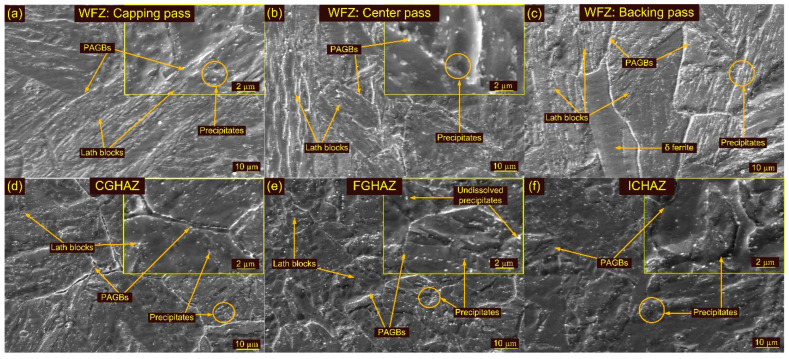
SEM images of weldments after HT1 treatment: WFZ (**a**) capping pass, (**b**) center pass, (**c**) backing pass; (**d**) CGHAZ, (**e**) FGHAZ, (**f**) ICHAZ.

**Figure 15:**
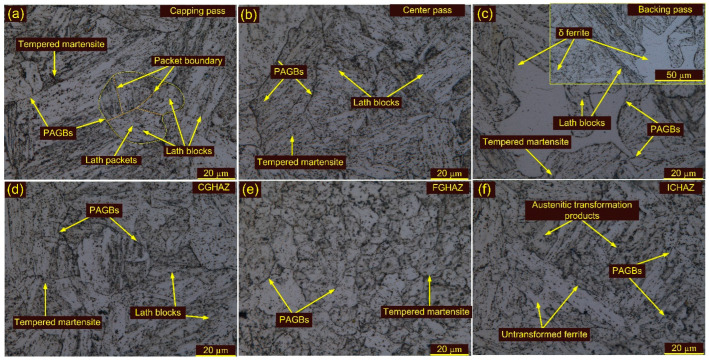
Weldments after HT2 treatment: WFZ (**a**) capping pass, (**b**) center pass, (**c**) backing pass; (**d**) CGHAZ, (**e**) FGHAZ, (**f**) ICHAZ.

Optical and SEM images of weldments after HT1 and HT2 treatment are presented in Figure 13, Figure 14 and Figure 15. The optical images in Figure 13a,c show the typical lath block-type microstructure in the capping and backing passes for HT1 treatment. However, lath morphology disappeared to some extent in the center pass (Figure 13b). The δ ferrite patches remained unaffected during HT1 and are seen in both the capping and backing pass regions (Figure 13c). The PAGs’ sizes in the capping, center and backing passes were 25 ± 5 µm, 38 ± 8 µm and 30 ± 6 µm, respectively. The SEM images corresponding to each pass are given in Figure 14a–c. The major precipitation is seen along the blocks and boundaries. The higher magnification image shows the decoration of the precipitates along blocks and boundaries and inside the matrix. Typical optical images of the HAZs are presented in Figure 13d–f. The HT1 resulted in a minute effect on the PAGs’ size in the CGHAZ, which was measured at 25 ± 6 µm. The FGHAZ showed an increase in PAG size, which was 13 ± 3 µm after HT1 treatment. The ATP and UF in ICHAZ still imparted a complex microstructure, marked in Figure 13f. The PAGs’ size was 12 ± 2 µm in the ICHAZ. SEM images of the HAZs are presented in Figure 14d–f. The CGHAZ exhibits the tempered martensitic microstructure with lath blocks of different orientations within lath packets. Coarse precipitates along lath blocks and PAGBs are observed from the SEM image (Figure 14d). An equiaxed grain structure consisting of lath blocks and boundaries was seen in the FGHAZ (Figure 14e). The major evolution of the precipitates is seen along blocks and boundaries, similar to other weldment regions. A complex microstructure still existed in the ICHAZ and showed random distribution of the precipitates within a tempered martensitic matrix. The difference in density between the carbide precipitates along the old PAGBs and those along the newly formed PAGBs is clearly distinguished from Figure 14f.

**Figure 16:**
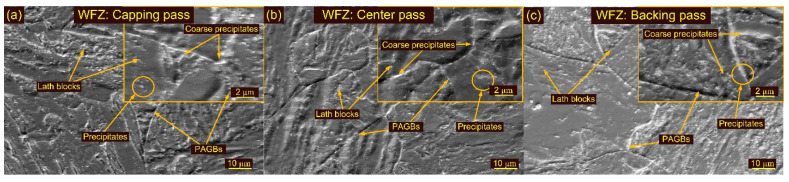
SEM images of weldments after HT2 treatment: WFZ (**a**) capping pass, (**b**) center pass, (**c**) backing pass.

Optical images of the WFZ after HT2 treatment are depicted in Figure 15a–c. The WFZ corresponding to the capping pass shows lath blocks and lath packets within the PAGBs. The lath blocks had different orientations within lath packets. Coarsening of the grains is also seen in the WFZ. The PAGs’ sizes in the capping, center and backing passes were 28 ± 5 µm, 40 ± 6 µm and 31 ± 4 µm, respectively. The WFZ corresponding to the backing pass showed typical lath blocks and δ ferrite patches (Figure 15c). SEM images are presented in Figure 16a–c. A higher magnification image presents the precipitate decoration along blocks and boundaries (Figure 16). The HAZs after HT2 are presented in Figure 15d–f. Coarse PAGs consisting of blocks and boundaries are seen in the CGHAZ (Figure 15d). The PAGs’ size was 32 ± 7 µm. Equiaxed PAGs with a size of 13 ± 3 µm are seen in the FGHAZ (Figure 15e). The ICHAZ after HT2 is presented in Figure 15f.

### 3.3. Mechanical Properties

**Figure 17:**
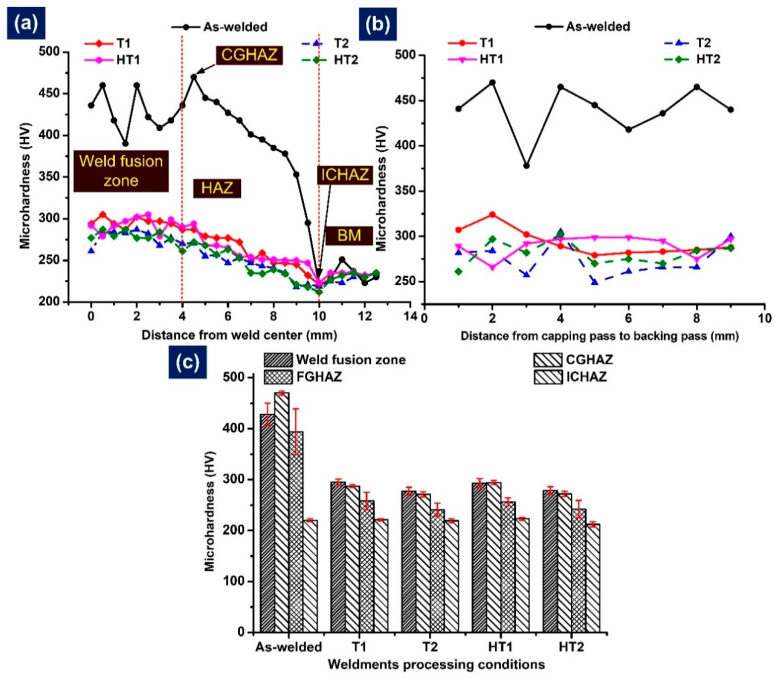
(**a**) Hardness profiles along the weldments, (**b**) hardness profiles, and (**c**) hardness of weldments after different heat treatment conditions.

The hardness variation plots for welded joints in AW and PWHT conditions are depicted in Figure 17a. In the AW condition, the hardness of the WFZ was 390–460 HV with an average of 428 ± 22 HV (Figure 17c). The variation in hardness might be due to the nature of multi-pass welding and the presence of soft δ ferrite patches [65]. The soft ferrite patches showed a hardness value much lower than that of the surrounding matrix of untempered martensite. Pandey et al. [49] also reported hardness values of δ ferrite patches between 179 and 301 HV. The hardness of the WFZ depends on heat input, filler composition and weight percentages of C and N in the solid solution matrix [66,67]. For a similar type of SMAW joint with P92 and P911 filler, the hardness of the WFZ was 571 ± 24 HV and 451 ± 20 HV, respectively [49]. For an autogenous TIG joint, the hardness of the WFZ was measured in a range of 404–480 HV [49]. The higher heat input in autogenous TIG led to the formation of ferrite patches, resulting in higher variation in the hardness value of the WFZ. The average hardness of the WFZ obtained in the case of the present joint was lower than the values reported for SMAW and autogenous TIG joints; however, variation in hardness value still existed due to the ferrite patches. The higher hardness of the WFZ might be due to the higher C and N contents and the formation of fresh martensite. A continuous reduction in hardness was measured along the HAZ as one moved along the BM from the fusion boundary. The hardness of the CGHAZ, FGHAZ and ICHAZ were 470 ± 4 HV, 394 ± 44 HV and 220 ± 3 HV, respectively. The CGHAZ hardness was measured to be higher than that of the WFZ. The CGHAZ was formed in the region of P92 BM; however, it offered hardness much higher than that of the BM (235 ± 4 HV). These results were attributed to higher C and N weight percentages in the CGHAZ solution matrix as it experienced the maximum temperature (above A_c3_) among the HAZs, resulting in complete dissolution of the carbide precipitates, as shown in Figure 5. Due to the higher hardness, the CGHAZ is considered a highly susceptible zone to hydrogen-induced cracking and offers poor impact toughness [19,68,69]. The FGHAZ was a wide region and showed a continuous reduction in hardness as one moved from the CGHAZ to the BM. The region of the FGHAZ near the CGHAZ experienced maximum temperature and showed a hardness of 445 HV, while nearer the ICHAZ, it experienced lower temperatures and showed a hardness of 295 HV. It is very clear that the hardness value of the FGHAZ was a function of the temperature experienced during the welding process. The average hardness of the FGHAZ was 394 ± 45 HV, which was lower than that of the CGHAZ. This might be due to the incomplete dissolution of the carbide precipitates in the FGHAZ, and it resulted in poor solid solution hardening. A sharp decline in hardness was noticed from the CGHAZ to the ICHAZ. The ICHAZ showed the lowest hardness among the HAZs, at 220 HV. The poor hardness of the ICHAZ makes it a crack-susceptible region during end service application, and in most published work, failure was reported in the ICHAZ during creep testing [70]. The ICHAZ experiences temperatures between the A_c1_ and A_c3_, which results in coarsening of M_23_C_6_ carbides and over-tempering of the martensite, in turn causing poor hardness. Previous studies also reported a similar hardness trend for P92 welded joints [65]. The effect of the welding passes on the hardness plot is shown in Figure 17b. The hardness in the capping pass and backing pass was measured to be higher than in the middle passes. This hardness variation exists due to the multi-pass nature, the heating effect and ferrite formation. The sudden drop in hardness near the capping pass (Figure 10b) was due to the δ ferrite patch. The lower hardness in the middle passes might be due to the heating effect of the backing and capping passes, which allowed for the tempering of the middle passes. Post-weld heating showed a drastic effect on the hardness of the weldments. PWHT is mainly conducted to lower the hardness variation and to enhance the performance of the weldments. The hardness plot along the transverse direction and thickness of weldments is depicted in Figure 17a,b. The hardness of the WFZ was 295 ± 6 HV, 277 ± 8 HV, 293 ± 9 HV and 278 ± 8 HV for T1, T2, HT1 and HT2, respectively. The drastic changes in the microstructure of WFZ, i.e., the tempering of martensite, lath breakup and evolution of precipitates, showed a considerable effect on the hardness of the WFZ. A 31% reduction after T1 and 35% reduction after T2 were measured for WFZ hardness. Similar reductions were observed after HT1 and HT2. A considerable hardness reduction was also measured for the CGHAZ, which was 287 ± 3 HV, 271 ± 5 HV, 294 ± 4 HV and 272 ± 5 HV for T1, T2, HT1 and HT2, respectively. Each region of the weldments showed a reduction in hardness except for the ICHAZ and BM. The hardness of ICHAZ was 221 ± 2 HV, 219 ± 4 HV, 223 ± 3 HV and 212 ± 5 HV for T1, T2, HT1 and HT2, respectively. However, variation in hardness still existed along the weldments. The difference between the maximum (CGHAZ) and minimum (ICHAZ) hardness values was 66 HV, 52 HV, 71 HV and 60 HV for T1, T2, HT1 and HT2, respectively. The PWHT at 120 min was more effective at minimizing the hardness variation. The reduction in hardness in the CGHAZ and FGHAZ is attributed to a tempering reaction [71]. The combined effect of PWHT and welding passes on hardness values is given in Figure 17b.

**Figure 18:**
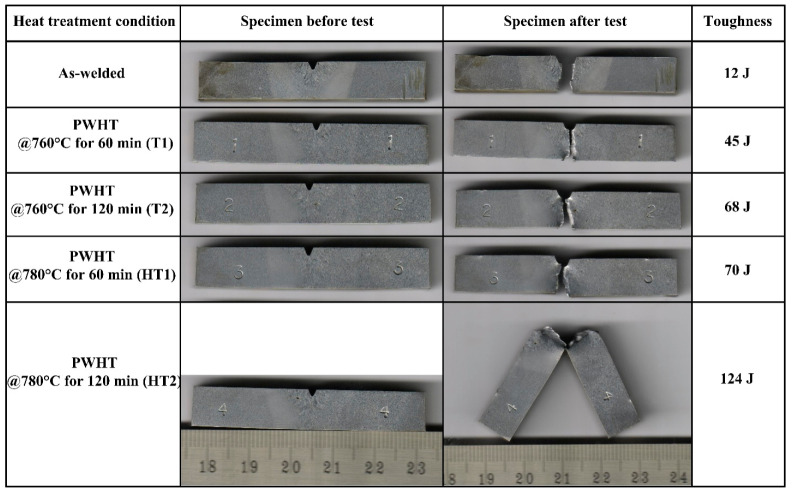
Impact test results for AW and heat-treated joints showing impact toughness values and impact specimens before and after fracturing.

To meet the essential requirements for the hydro testing of vessels, the minimum impact toughness of the WFZ was recommended to be 47 J (EN ISO 3580:2017) [72]. The ASME standard also recommended that the minimum impact toughness value of the WFZ should be more than 41 J. The impact toughness of the WFZ was 12 J in the AW joint, which was lower than the minimum required value. The impact specimen before and after fracture is given in Figure 18. The fractured specimen for the AW joint supports the impact test results, as there was a complete fracture of the specimen into two parts without any shear deformation or yielding. The impact toughness of the P92 HAZ and BM were 65 J and 152 J, respectively. In Cr–Mo weldments, different zones have different microstructural features, resulting in varying mechanical properties, i.e., impact toughness and hardness [68]. The P92 HAZ impact toughness was measured to be lower than that of the P92 BM but higher than that of the WFZ. The WFZ was observed to be the weakest portion of the welded joint in terms of impact toughness. The poor impact toughness of WFZ might be due to its untempered lath martensitic microstructure, which imparts brittleness to the WFZ. The poor impact toughness of the WFZ might also be due to the presence of the ferrite stabilizer in higher weight percentages [65]. The impact toughness difference between the highest (P92 BM) and lowest (WFZ) values was 143 J. However, the impact toughness of the WFZ was measured to be higher than an A-TIG joint of P92 steel, which might be due to the presence of soft ferrite patches in the WFZ of the A-TIG joint [49]. For a SMAW joint of P92 steel, the impact toughness of the WFZ was measured to be 7 ± 2 J by Saini et al. [18]. Hence, it can be stated that the impact toughness of the WFZ obtained for the multi-pass GTAW joint was better than that of the A-TIG and multi-pass SMAW joints.

For the rest of the weldment conditions, impact toughness was measured for the WFZ only and the results are presented in Figure 18. T1 improved impact toughness significantly, as it imparted ductility to the WFZ by tempering the martensite. However, impact toughness still measured lower than the minimum accepted value of 47 J. After T2, i.e., at a constant temperature with increased PWHT time from 90 min to 120 min, impact toughness was 68 J, close to the recommended value of 47 J. The increase in impact toughness was attributed to the tempering reaction. Tempering of martensite imparts softening to the martensitic matrix via a reduction in dislocation density, breakup of lath and evolution of precipitates. In both T1 and T2 treatment, a complete fracture of the test specimen occurred, which ensured that the mixed-mode fracture was dominated by a cleavage area or brittle fracture. With the increase in PWHT temperature, i.e., from 760 °C (T1) to 780 °C (HT1) for a constant PWHT duration of 90 min, impact toughness increased from 45 J to 70 J and achieved the minimum landmark of 47 J. That result might be due to adequate tempering of the martensite. The impact test results for HT2 showed the highest impact toughness value of 124 J (Figure 18). The test specimen for HT2 also showed yielding before fracture, as given in Figure 18. A great impact of PWHT parameters on the impact toughness of Cr–Mo steel welded joints was also reported by Pandey and Mahapatra [69]. From the impact test results, it can be recommended to either perform PWHT at 760 °C for 120 min (T2) or at 780 °C for 90 min (HT1). T1 resulted in poor impact toughness while HT2 needed a higher cost than HT1.

**Figure 19:**
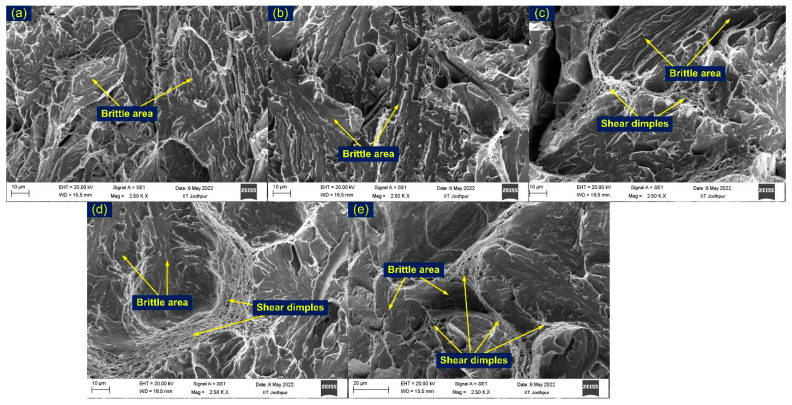
SEM image of fractured impact tested specimens: (**a**) AW, (**b**) T1, (**c**) T2, (**d**) HT1, (**e**) HT2.

SEM micrographs of the impact-tested fractured specimens are presented in Figure 19. The appearance of the fracture surface for AW and T1 specimens supports the impact test results, as in both cases the fracture was dominated by a brittle area (Figure 19a,b). The fracture surface shows the typical characteristics of a cleavage area and river patterns. In T2, both dimples and a cleavage area appeared at the fracture surface; however, the fracture mode was dominated by the brittle area (Figure 19c). In HT1 (Figure 19d) and HT2 (Figure 19e), both cleavage and shear dimples are seen; however, the major portion was represented by the brittle area.

**Figure 20:**
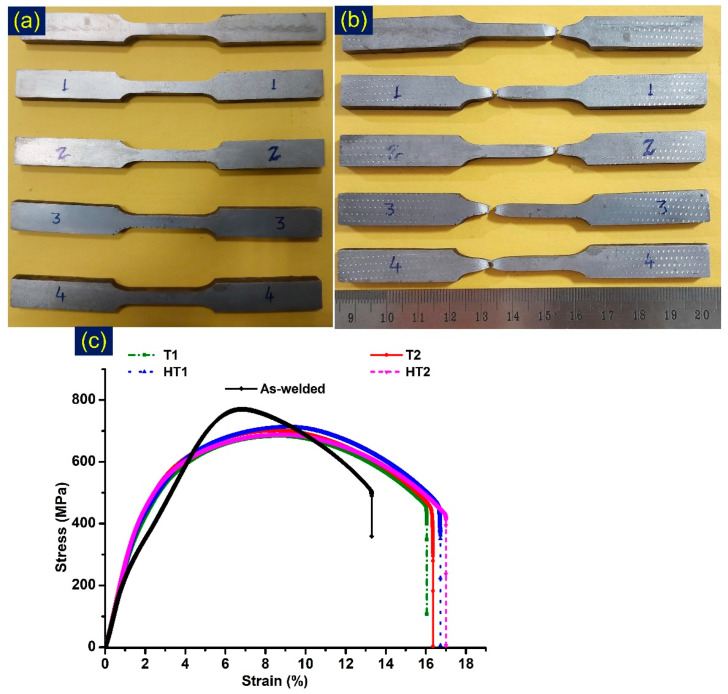
(**a**) Tensile specimens before testing and (**b**) after testing; (**c**) stress–strain curve for tested specimens.

Tensile test results are depicted in Table 4. Tensile specimens before and after fracture are given in Figure 20a,b, while Figure 20c shows the stress–strain curve. The specimen for the AW test showed the fracture in the P92 BM zone with tensile strength (TS) of 751 MPa. The failure of the tensile specimen from the P92 BM instead of the WFZ ensured that the joint was safe for USC boiler application. The TS value measured near the P92 BM was 758 MPa. However, for the AW joint, % elongation (13%) measured lower than that of the P92 BM (33%). The lower value might be due to the presence of the WFZ and HAZ in the gauge area of the specimen. The TS values were 683 MPa, 701 MPa, 711 MPa and 690 MPa for T1, T2, HT1 and HT2, respectively. The fracture in each condition was measured in over-tempered BM. TS for the heat-treated specimens measured lower than for the BM and AW joint. This might be due to the over-tempering of the martensite in the P92 BM.

## 4. Conclusions

In the present work a systematic investigation of the influence of PWHT parameters on the mechanical properties and microstructural behavior of multi-pass GTAW joints of P92 steel was performed. The following conclusions were drawn:

1. A microstructural study along the weldments indicated that the as-welded joint had a significant amount of nonuniformity in terms of grain size, precipitate size and distribution. Each region along the weldments showed a specific microstructural feature. The complete dissolution of the precipitates was seen in the WFZ and CGHAZ, while a partial dissolution was observed in the FGHAZ. Coarse PAGs were seen in the CGHAZ and WFZ, while minimum PAG size was measured in the ICHAZ. The variation in microstructure resulted in nonuniformity in the mechanical properties.
2. Heterogeneity in the microstructure of the WFZ also existed due to the effects of welding passes and variations in cooling rate. The WFZ corresponding to the capping and backing passes showed the presence of soft δ ferrite patches, which offered poor hardness compared to the surrounding untempered martensitic matrix and resulted in heterogeneity in the microstructure and mechanical properties. The effect of the welding passes was also reflected in grain size.
3. PWHT resulted in a major observed effect on the WFZ and CGHAZ, as in both regions dissolution of the carbide precipitates occurred during welding. The PWHT treatment reduced nonuniformity along the weldments via a tempering reaction. The precipitates dissolved during welding in the CGHAZ, FGHAZ, and WFZ. However, nonuniformity in grain size still existed along the weldments. Treatments T1 and HT1 had a minute effect observed on the grain size in the HAZs; however, T2 and HT2 showed a considerable change in grain size in a few HAZ regions.
4. A hardness gradient existed along the weldments after welding and the difference between maximum hardness (CGHAZ) and minimum hardness (ICHAZ) was 208 HV. The peak hardness in the CGHAZ was attributed to precipitate dissolution, while the minimum hardness in the ICHAZ was attributed to partial tempering and precipitate coarsening. PWHT reduced the hardness gradient. The T2 and HT2 were observed to be more effective treatments to minimize the hardness gradient. The difference between maximum hardness (CGHAZ) and minimum hardness (ICHAZ) was 52 HV and 60 HV for T2 and HT2, respectively.
5. The tensile test results showed that the welded joint failed from the P92 BM for each condition, which ensured that the welded joint was safe for application. The tensile strength for the as-welded joint was close to that of P92 BM, while after PWHT, the tensile strength of the joints was reduced, even though the failure was still in the P92 BM region. This was attributed to tempering of the martensite.
6. The impact test results showed poor energy absorbing capacity of the weldments without heat treatment. The impact toughness of the WFZ was 12 J, due to the presence of a brittle martensitic phase. A significant improvement in impact toughness occurred after PWHT. However, for T1, impact toughness (45 J) still measured close to the minimum recommended value of 47 J. The best value of 124 J was measured for HT2.
7. To achieve the optimal microstructural and mechanical properties, the recommended PWHT temperature and time were 760 °C and 120 min, respectively, for a multi-pass GTAW joint of P92 steel.

## Figures and Tables

**Table 1:** Chemical composition of P92 steel, P92 (BOHLER P92-IG) filler and weld metal (wt.%).

Element	P92 Steel	Filler Metal	Weld Metal
C	0.092	0.11	0.10
Si	0.21	0.24	0.22
Mn	0.42	0.51	0.45
Cr	8.52	8.80	8.68
Mo	0.45	0.50	0.52
W	1.94	1.60	1.72

**Table 2:** Welding parameters.

Welding Pass	Welding Current (A)	Arc Voltage (V)	Travel Speed (mm/min)	Heat Input (kJ/mm)
First pass (root pass)	130	15.4	85	0.848
Second pass (filling pass)	120	14.8	80	0.799
Third pass (capping pass)	120	14.8	80	0.799
Fourth pass (backing pass)	120	14.8	90	0.710

**Table 3:** Post-weld heat treatment (PWHT) parameters.

Weld Plates	Temperature	Time	Specimen
As-welded	-	-	AW
	760 °C	90 min	T1
	760 °C	120 min	T2
PWHT	780 °C	90 min	HT1
	780 °C	120 min	HT2

**Table 4:** Tensile properties of P92 steel and welded joint for AW and heat-treated state.

Properties	Units	P92 Steel	As-Welded	T1	T2	HT1	HT2
Tensile strength	MPa	758	751	683	701	711	690
Elongation	%	33	13	16	16	17	17
Fracture location	-	-	P92 BM	P92 BM	P92 BM	P92 BM	P92 BM

## Data Availability

Not applicable.
